# Sodium‐glucose transport protein 2 inhibitor use in the management of insulin dysregulation in ponies and horses

**DOI:** 10.1111/jvp.13470

**Published:** 2024-07-10

**Authors:** Nicola J. Menzies‐Gow, Edward J. Knowles

**Affiliations:** ^1^ Department of Clinical Science and Services Royal Veterinary College Hertfordshire UK

**Keywords:** equine, gliflozin, glucose, insulin, laminitis, SGLT2i

## Abstract

Laminitis is a common and painful condition of the equine foot and approximately 90% of cases are associated with insulin dysregulation (ID) that is a central feature of the common endocrine disorder equine metabolic syndrome (EMS) and occurs in a subset of animals with pituitary pars intermedia dysfunction. Additional features of EMS include obesity, altered circulating concentrations of adipokines (particularly adiponectin and leptin) and hypertriglyceridaemia. Obesity, ID, hypoadiponectinaemia, hyperleptinaemia and an altered plasma lipid profile are also features of human metabolic syndrome (HMS) alongside hyperglycaemia. Sodium‐glucose cotransporter 2 inhibitors (SGLT2i) are a novel class of oral hypoglycaemic agents used in combination with lifestyle changes in the management of HMS. SGLT2 receptors are responsible for 90% of the renal glucose reabsorption that occurs in the proximal convoluted tubule. Thus, these drugs increase urinary glucose excretion by suppressing glucose reabsorption from the glomerular filtrate resulting in urinary calorie loss with consequent weight loss and improvements in ID, hyperglycemia, hypoadiponectinaemia and hyperleptinaemia. There are no licenced veterinary drugs available for treating ID and preventing insulin‐associated laminitis in horses. Thus, the use of SGLT2i for the control of equine hyperinsulinaemia with the goal of improving recovery from associated active laminitis or preventing future laminitis has recently been advocated. There are a small number of published studies reporting the use of the SGLT2i canagliflozin, ertugliflozin and velagliflozin to aid the management of equine ID. However, the doses used are largely extrapolated from human studies with limited consideration of species‐specific variations. In addition, there is limited evaluation of the fundamental differences between ID in horses and humans, particularly the fact that most horses with ID remain hyperinsulinaemic but normoglycaemic such that increased urinary loss of glucose may not explain the beneficial effects of these drugs. Further study of the potential deleterious effects of treatment‐associated hypertriglyceridaemia is required together with the effect of SGLT2i therapy on circulating concentrations of adipokines in horses.

## 1 INTRODUCTION

### 1.1 Equine laminitis

Laminitis is a common and painful condition of the equine foot that causes significant morbidity and mortality and its management poses a daily challenge in equine practice (Ireland et al., 2011). Although over 80 potential causes for laminitis have been proposed (Heymering, 2010), there are considered to be three main forms of laminitis, namely sepsis‐associated laminitis that occurs secondary to SIRS and/or sepsis, supporting limb laminitis that occurs in horses with severe lameness in the contralateral limb, and endocrinopathic laminitis that encompasses laminitis associated with insulin dysregulation (ID; Patterson‐Kane et al., 2018).

Endocrinopathic laminitis is the most common form of laminitis, accounting for up to 90% of cases of laminitis in some studies (Karikoski et al., 2011). It incorporates laminitis associated with ID, which is a central feature of the common endocrine disorder equine metabolic syndrome (EMS) and occurs in around 25%–50% of animals with pituitary pars intermedia dysfunction (PPID) (Patterson‐Kane et al., 2018). Obesity, altered circulating concentrations of the adipose tissue‐derived hormones adiponectin and leptin, and hypertriglyceridaemia are additional features of EMS (Durham et al., 2019).

### 1.2 Equine insulin dysregulation

Insulin dysregulation is defined as abnormal insulin metabolism in response to a normal physiologic process such as eating. In horses and ponies, it manifests in three forms, namely basal hyperinsulinaemia in which circulating insulin concentrations are consistently high; an excessive insulin response to carbohydrates in which ingestion of sugar or starch results in abnormally high circulating insulin concentration peaks; and tissue (peripheral) insulin resistance in which the resistance is at the level of the tissues (Durham et al., 2019). Any combination of these three forms can exist in an individual animal. The vast majority of horses and ponies with ID remain normoglycaemic, with only rare cases of diabetes mellitus reported in the scientific literature (Durham et al., 2009; Giri et al., 2011; Jeffrey, 1968; Johnson et al., 2005; Newman, 2015).

The exact pathogenesis of equine ID associated with EMS remains unknown, but there is evidence that genetics (Lewis et al., 2017; Treiber, Kronfeld, Hess, et al., 2006), epigenetics (Robles et al., 2017, 2018), obesity (Kearns et al., 2006; Morgan et al., 2014), diet (Pratt et al., 2006), the microbiome (Biddle et al., 2018) and endocrine disrupting chemicals may play a role (Durward‐Akhurst et al., 2017). The mechanism for any associations between PPID and ID has also yet to be determined. It is postulated that increased production of some of the pars intermedia‐derived hormones plays a role; for example, corticotropin‐like intermediate peptide (CLIP) stimulates insulin secretion (Marshall et al., 1984) whilst β‐cell tropin, a breakdown product of CLIP, is also an insulin secretagogue and activates endogenous insulin (Watkinson & Beloff‐Chain, 1984). Alternatively, in some cases, the PPID may exacerbate existing ID through yet undetermined processes (Tadros et al., 2019). Additionally, recent evidence suggests that associations between PPID and ID may be seasonally dependent (Knowles et al., 2024; Li et al., 2023).

### 1.3 Association between ID and laminitis

It is well‐established that there is a link between hyperinsulinaemia and laminitis in horses and ponies (Durham et al., 2019). Field‐based studies have shown an association between hyperinsulinaemia, or other components of ID, and laminitis (Jeffcott et al., 1986; Treiber, Kronfeld, & Geor, 2006) and experimental studies demonstrated that laminitis can be induced by 48–72 h of insulin infusion in healthy ponies whilst maintaining euglycaemia (Asplin et al., 2007) and horses (de Laat et al., 2010). More recently it has been demonstrated that ID is an independent risk factor for endocrinopathic laminitis (Knowles et al., 2023; Menzies‐Gow et al., 2017) and ID is a feature of the subset of animals with PPID that develop laminitis (Horn et al., 2019; Karikoski et al., 2016; McGowan et al., 2013). However, exactly how hyperinsulinaemia causes laminitis remains to be determined. As experimental evidence has advanced, various plausible theories have been undermined including glucose deprivation (Asplin et al., 2011), glucotoxicity (de Laat, Kyaw‐Tanner, et al., 2012), matrix metalloprotease upregulation (de Laat et al., 2011) and increased blood flow‐induced hyperthermia (de Laat, Pollitt, et al., 2012). Current theories with some supporting evidence include endothelial dysfunction resulting in vasoconstriction (Morgan et al., 2016; Venugopal et al., 2011; Wooldridge et al., 2014), inappropriate signalling via binding of insulin to lamellar insulin‐like growth factor receptors (Burns et al., 2013; Lane et al., 2017; Nanayakkara et al., 2019) and gut microbiota and gut permeability changes (Elzinga et al., 2016; Staniar et al., 2016). None of these theories have yet to be definitively proven and there are conflicting data relating to many of these theories such as the development of laminitis without evidence of impaired microvascular perfusion or negative energy balance (Stokes et al., 2020) and the apparently poor binding affinity between insulin and lamellar IGF receptors (Nanayakkara et al., 2019).

### 1.4 The inter‐relationship between equine ID, obesity, adipokines and laminitis

Whilst ID is the central feature of ID, obesity and altered circulating concentrations of the adipose tissue‐derived hormones adiponectin and leptin, as well as hypertriglyceridaemia, are additional features of EMS and EMS is associated with an increased risk of endocrinopathic laminitis (Durham et al., 2019). Thus, there is a complex inter‐relationship between these factors.

Obesity is associated with an increased risk of laminitis (Carter, Treiber, et al., 2009) and an overweight or obese horse or pony is nine times more likely to have hyperinsulinaemia than a moderately conditioned animal (Carter, Geor, et al., 2009). In addition, a possible consequence of the increased fat mass is dysregulation of adipokine production, including adiponectin and leptin (Radin et al., 2009; Selim et al., 2015).

Leptin serves to relay signals to the brain indicating fat status and to maintain body condition by appetite suppression and increased energy expenditure during energy excess (Van Weyenberg et al., 2013). Adipocytes are the main site of leptin synthesis and fat mass appears to be the primary determinant of serum leptin concentration in the horse (Kearns et al., 2006). In some horses leptin concentrations exceed those expected based on the amount of fat due to the presence of obesity‐related leptin resistance, which may act to worsen the existing degree of obesity (Kearns et al., 2006; Van Weyenberg et al., 2013). Increased leptin concentrations have also been correlated with hyperinsulinemia in ponies (Morgan et al., 2014) and were beneficial in the prediction of laminitic episodes in a cohort of Welsh and Dartmoor ponies (Carter, Treiber, et al., 2009).

Adiponectin has anti‐inflammatory properties and is insulin sensitizing (Stefan et al., 2002; Waki et al., 2003). Hypoadiponectinaemia is an independent risk factor for the development of endocrinopathic laminitis (Knowles et al., 2023; Menzies‐Gow et al., 2017; Wray et al., 2013) and adiponectin concentrations are weakly positively associated with obesity in some equine studies (Barnabe et al., 2024) but negatively correlated in others (Kearns et al., 2006; Wooldridge et al., 2012). Additionally, horses with ID have lower adiponectin concentrations than horses without ID (Fitzgerald et al., 2019; Karikoski et al., 2022; Wooldridge et al., 2012).

## 2 RATIONALE FOR SGLT2i USE IN THE MANAGEMENT OF EQUINE ID

Obesity, ID, hypoadiponectinaemia, hyperleptinaemia and an altered plasma lipid profile are features of human metabolic syndrome (HMS) (Reaven, 2002) as well as EMS. Sodium‐glucose transport protein 2 inhibitors (SGLT2i) are a novel class of oral hypoglycemic agents used in combination with lifestyle changes in the management of HMS. SGLT2 receptors are responsible for 90% of the renal glucose reabsorption that occurs in the proximal convoluted tubule. Thus, these drugs increase urinary glucose excretion by suppressing glucose reabsorption from the glomerular filtrate resulting in urinary calorie loss with consequent weight loss and improvements in ID, hyperglycemia, hypoadiponectinaemia and hyperleptinaemia (Wang & Xia, 2022; Wu et al., 2019). The overall result is significant cardiorenal protective effects (Akiyama et al., 2023). Whilst some of these beneficial effects can be explained by improved glycaemic control, other mechanisms are thought to be involved. Early natriuresis with a reduction in plasma volume, a consequent rise in haematocrit, improved vascular function, a reduction in blood pressure and changes in tissue sodium handling are all likely to have a role (Cowie & Fisher, 2020). Additional mechanisms of SGLT2i that might be beneficial include a reduction in adipose tissue‐mediated inflammation and pro‐inflammatory cytokine production, a shift towards ketone bodies as the metabolic substrate for the heart and kidneys, reduced oxidative stress, lowered serum uric acid level, reduced glomerular hyperfiltration and albuminuria, and suppression of advanced glycation end‐product signalling (Cowie & Fisher, 2020).

There are no licenced veterinary drugs available for treating ID and preventing insulin‐associated laminitis in horses. Thus, the use of SGLT2i for the control of equine hyperinsulinaemia with the goal of improving recovery from associated active laminitis or preventing future laminitis has recently been advocated (Frank, 2018; Kellon & Gustafson, 2022; Sundra et al., 2022). If SGLT2i use in the horse was also associated with weight loss and improvements in hypoadiponectinaemia and hyperleptinaemia as occurs in people, then these consequences would likely contribute to reducing the risk of future laminitis. Further research is required to determine whether these improvements do indeed occur in the horse.

A further potential but as yet unreported indication for SGLT2i use in the horse may be as an enablers of corticosteroid treatment. Corticosteroid medication is commonly used for orthopaedic indications in the horse and induces transient increases in serum insulin concentration (Boger et al., 2024). In the vast majority of cases, corticosteroid treatment is considered safe but there is a risk that consequent increased serum insulin concentrations may precipitate laminitis in predisposed individuals (Potter et al., 2019). SGLT2i have been proposed as enablers of corticosteroid treatment in human patients (Kahwash & Butler, 2023) and further research is required to determine whether this approach is valid in the horse.

## 3 SGLT2i THAT HAVE BEEN USED IN THE HORSE AND RELATIVE SGLT1/2 SELECTIVITY

SGLT2i that have been reportedly used in the horse to date include the gliflozins velagliflozin (Meier et al., 2018, 2019), canagliflozin (Frank, 2018; Kellon & Gustafson, 2022; Lindase et al., 2023; Michanek et al., 2023), ertugliflozin (Sundra et al., 2022; Sundra, Rossi, et al., 2023) and dapagliflozin (anecdotal reports). All of these are used in humans apart from velagliflozin, which was recently licenced for the treatment of diabetes mellitus in cats not previously treated with insulin (Hoenig et al., 2018). It is difficult to estimate the scale of current SGLT2i use in the horse. Anecdotal reports suggest widespread clinical use, particularly in the United Kingdom and Australia that has preceded much of the current, limited, equine scientific literature. UK sales of over an estimated 220,000 individual doses of an extemporaneous preparation of ertugliflozin have been reported by one manufacturer (Jones, E. personal communication). Given that other SGLT2i preparations (such as tablet forms of ertugliflozin and canagliflozin) are also known to be used, the total equine market size is likely to be considerable.

Receptor selectivity of SGLT2i varies greatly between agents; most of those used in horses are between 250‐ and 2500‐fold more selective for SGLT2 over SGLT1 receptors (Cinti et al., 2017), however, the fold receptor selectivity of velagliflozin is not reported. It is also unknown whether the receptor selectivity shown in other species is equivalent in the horse. Meta‐analysis of human studies has demonstrated that all SGLT2i improve fatal and non‐fatal cardiac events when compared to placebo; however, the cardiac benefits of SGLT2i increase with decreasing receptor selectivity, with the non‐selective agent sotagliflozin showing superior efficacy (Tager et al., 2022). In contrast, receptor selectivity does not affect mortality or renal endpoints and no significant difference between individual SGLT2i was noted (Tager et al., 2022). Finally, for end‐points more directly associated with diabetes meta‐analyses of randomized clinical trials have demonstrated that all four SGLT2i available for use in people achieve similar outcomes with respect to glycaemic control with weight loss and without increasing hypoglycaemia as monotherapies (Tentolouris et al., 2019). Different studies have not shown a consistent advantage to one particular SGLT2i over others. In one meta‐analysis improvements were greater with canagliflozin 300 mg monotherapy than with other SGLT2i treatments or canagliflozin 100 mg (Shyangdan et al., 2016). In another meta‐analysis, ertugliflozin was more effective than both dapagliflozin and empagliflozin (more and less selective agents) for improving glycaemic control when combined with diet and exercise (McNeill et al., 2019).

There is no evidence available to suggest that any individual SGLT2i is associated with a better outcome in horses with ID, mainly due to the lack of published evidence. In addition, whilst the pharmacokinetics and pharmacodynamics of available SGLT2i for use in humans have been extensively studied, only the pharmacokinetics of velagliflozin in cats (Anon, 2024) and of ertugliflozin in dogs (Fediuk et al., 2020) are reported in the peer‐reviewed scientific literature. Limited pharmacokinetic/pharmacodynamic data for velagliflozin use in horses was included in a publicly available patent application (Reiche et al., 2015). Horses (*n* = 3 per group) received a single dose of velagliflozin at 1 mg/kg i. v, 0.3 mg/kg p.o. or 3 mg/kg p.o. The plasma half‐life was estimated at 7.9–8.5 h and Tmax following oral dosing occurred at 1 h for the 3 mg/kg dose and 2 h for the 0.3 mg/kg dose. Equine pharmacokinetic data for the SGLT2i preparations used most commonly in equine clinical practice (ertugliflzon, canagliflzin and dapagliflzin) have not been reported. Such use is therefore largely extrapolated from human studies with limited consideration of species‐specific variations in important areas such as bioavailability, therapeutic plasma concentrations, excretion rates or toxicity. For example, the reported plasma half‐life of velagliflozin (8 h) is shorter than half‐lives commonly reported for other SGLT2i in human studies of 11–13 h (Patel & Nair, 2022). Whilst further investigation is required these data may suggest an indication for a shorter dosing interval in (some) horses.

Importantly, at this stage in the clinical use of SGLT2i in the horse, much of the published data reports uncontrolled, unblinded clinical case series. Whilst such studies may have the advantage of representing ‘real world’ clinical conditions, they include a risk of bias including the potential effects of concurrent treatment and management, optimism bias and placebo effects that should be considered when interpreting the study findings.

## 4 USE OF SGLT2i IN HORSES IN THE SCIENTIFIC LITERATURE

### 4.1 Canagliflozin

Experimental use of canagliflozin was first reported in a study of six horses with ID treated for 3 days (Frank, 2018). Canagliflozin was reported to lower serum insulin concentration following an oral sugar test (OST) when given at ‘medium’ and ‘high’ doses, but dosage were not reported. No adverse health effects were observed, and serum triglyceride concentrations were not reported. In a second experimental study, two standardbred mares received canagliflozin at relatively high doses of 1.8 and 3.6 mg/kg po. Treatment reduced serum insulin and glucose concentrations in response to a graded glucose infusion (Michanek et al., 2023).

The first use of an SGLT2i in equine clinical practice was reported in a case series. Ten horses with hyperinsulinaemia refractory to dietary control and pharmacologic management of the ID and PPID were treated with the SGLT2i canagliflozin (0.3–0.6 mg/kg once daily orally); nine of these animals were also hyperglycaemic (Kellon & Gustafson, 2022). Treatment corrected hyperglycaemia, reduced insulin to normal (7/10) or near normal (3/10) concentrations and was 100% effective in reversing or reducing abnormal fat pads and eliminating laminitis pain; however, increases in insulin were observed when the concomitant PPID was not controlled, or diet was liberalized. Thus, the authors recommend that the core aspects of therapy—diet control, exercise when possible and adequate treatment of PPID—must also be maintained if using canagliflozin (Kellon & Gustafson, 2022).

In a second retrospective case series, the use of canagliflozin in the treatment of 17 horses and ertugliflozin in a further three horses with refractory hyperinsulinaemia were described (Kellon & Gustafson, 2023). Animals were concurrently meant to be fed a low carbohydrate forage diet. After the initiation of SGLT2i, mean glucose decreased by 18.8% from the pre‐intervention baseline and remained stable at the second retest with only a 1.5% decrease from the first retest. Serum insulin (presumed to be basal measurements) decreased by 48.5% between the baseline and first retest and there was a 6.9% decrease between the first and second retest. Triglycerides were low before initiating drug therapy, but at the first retest, triglycerides increased significantly by 256.1% over the pre‐treatment baseline. At the second retest, triglycerides decreased by 38% when compared to the first retest period. In cases where diet and PPID were not fully controlled, the result was an increase in insulin concentration. As doses were decreased due to high triglycerides, insulin increased and doses below 0.3 mg/kg appeared to be ineffective for insulin control. However, canagliflozin administered at 0.6 mg/kg appeared to result in a greater increase in triglyceride concentration. Nineteen of 20 treated horses were reported sound, despite variable individual insulin responses to the drug and many animals having insulin concentrations above normal. One horse developed laminitis despite good insulin control. The time to resolution of laminitis lameness ranged from 24 h to several weeks, likely due to individual differences in the degree of chronic laminitic hoof changes such as rotation and/or sinking of the distal phalanx as well as the adequacy of hoof care. The authors concluded that the significant increase in triglycerides and reported laminitis relief associated with SGLT2i therapy deserves further investigation and that carefully designed controlled trials will help to determine ideal dosages and optimal diet, develop strategies to control hypertriglyceridemia, and explore potential drug mechanisms behind the relief of laminitis.

In a double‐blinded, randomized placebo‐controlled trial, the short‐term effect of canagliflozin (0.6 mg/kg once daily orally for 3 weeks) versus placebo on glucose and insulin responses to an OST as well as the effects on body weight and triglyceride concentrations were compared in 16 horses with ID but normoglycaemia (Lindase et al., 2023). Treatment with canagliflozin significantly decreased the insulin response to an OST; however, only 50% of the treated horses had normal insulin concentrations after the OST. In addition, the insulin response decreased to a greater extent than the glucose response, thus the treatment effect of decreasing the insulin response may not be attributed solely to a decrease in the glycemic response but may instead be associated with a treatment‐associated decrease in pancreatic β‐cell responsiveness to glucose (Lindase et al., 2023). The canagliflozin‐treated horses also experienced greater weight loss over the treatment period compared to those treated with placebo.

### 4.2 Dapagliflozin

Whilst there are anecdotal reports of using dapagliflozin to aid the management of ID in horses, there is no available scientific evidence.

### 4.3 Ertugliflozin

Preliminary observations on the use of ertugliflozin in the management of hyperinsulinaemia and laminitis in 51 horses have been reported (Sundra et al., 2022) and 0.05 mg/kg once daily orally was associated with a marked reduction in circulating insulin concentrations and lameness grade associated with laminitis at 30 days. Insulin concentrations remained low through subsequent follow‐up tests in most horses, but in 8/51 (16%), an increase in insulin was identified beyond 30 days of treatment, potentially due to a compensatory role of renal SLGT1 receptors or due to owners failing to adhere to the concurrent long‐term diet and management recommendations. Although most horses lost weight whilst they were on treatment with ertugliflozin, rates of weight reduction were variable and a lack of an untreated control group means that further investigation is required to determine whether it offers a means of promoting the reduction in adiposity beyond what can be achieved with dietary restriction alone. The dose used reflected the dose that is being used in equine practice based on publications from other species and anecdotal experience, it does not have an evidential basis in the horse. The authors commented that pharmacokinetic and pharmacodynamic studies are warranted to establish the most appropriate dosing regimen for this drug in horses. It should also be noted that the pre‐ or post‐treatment blood glucose concentrations of the animals included in the study were not reported.

In a second study, clinical records were reviewed to identify horses with ID that had an OST performed before and after 4 days of treatment with ertugliflozin (Sundra, Rossi, et al., 2023). Ten horses met the inclusion criteria and four daily doses of ertugliflozin at 0.05 mg/kg once daily orally were associated with lowering insulin concentrations at baseline and in response to an OST; however, insulin concentrations did not return to normal in all horses. It should be noted that none of the horses were hyperglycaemic prior to treatment.

### 4.4 Velagliflozin

Velagliflozin is currently only commercially available in an oral formulation for cats; however, it has been used in equine experimental studies. Limited details of some of these experimental studies are reported in a patent specification (Reiche et al., 2015) including estimated plasma half‐life and t‐max (described above). Horses that received velagliflozin developed glucosuria within an hour of administration (at 1 mg/kg i.v., 0.3 or 3 mg/kg p.o.) but maintained euglycaemia even at high doses (3 mg/kg p.o.) Following a 4‐week, placebo‐controlled trial treatment (*n* = 4 control and *n* = 4 treated horse (0.1–1 mg/kg p.o. sid)) glucose and insulin responses to an OST were reduced, non‐esterified fatty acid clearance was increased and plasma leptin concentrations and body weight reduced (Reiche et al., 2015). Although relatively limited data are available the range of doses used in these studies suggests a relatively wide therapeutic index for velagliflozin. Treatment with velagliflozin (0.3 mg/kg once daily orally for 3 weeks) also lowered post‐prandial insulin and glucose concentrations and prevented laminitis development in 12 ponies with ID but normoglycaemia fed a challenge diet high in non‐structural carbohydrates for up to 18 days compared to 37 ponies with ID and normoglycaemia that were not treated (Meier et al., 2018). Although in this study there was no significant change in weight in any of the ponies over the study period. In a second study, 24 ponies with ID and normoglycaemia were treated with velagliflozin at the same dose for 16 weeks and fed a maintenance diet (rather than a high carbohydrate diet) (Meier et al., 2019). Post‐prandial serum insulin concentrations were significantly decreased at week 16, but not week 8, and had returned to pre‐treatment values 4 weeks after withdrawal of treatment with no rebound effect. Body weight remained unchanged in all animals over the study period.

## 5 SIDE EFFECTS OF SGLT2i IN HORSES

### 5.1 Hypertriglyceridaemia

There are some important differences in the metabolic alterations that occur in association with SGLT2i therapy between horses and people treated with SGLT2i. In people, SGLT2i shift substrate utilization from carbohydrates to lipids and affect the plasma lipid profile, seen as significantly increased total cholesterol, LDL‐cholesterol, non‐HDL‐cholesterol and HDL‐cholesterol, and decreased triglyceride concentrations (Tentolouris et al., 2019). In addition, in people SGLT2i are ketogenic, however in most cases the increases in serum ketones are mild and may be beneficial (Koutentakis et al., 2023). Rarely SGLT2i may cause the serious complication of euglycaemic diabetic ketoacidosis (euDKA) in diabetic people (Wiviott et al., 2019) and in cats (Hadd et al., 2023). The pathophysiology of euDKA in patients receiving SGLT2i remains unclear, but there are multiple hypotheses explained to understand the mechanism of euDKA. The most popular hypothesis was that SGLT2i increase glucagon secretion by binding to alpha cells of pancreatic islets and causing gluconeogenesis (Wibawa et al., 2021). Simultaneously, it decreases blood glucose by hastening its renal excretion from the plasma. A decrease in blood glucose causes decreased insulin secretion from islets which results in excess ketone body formation. Hypertriglyceridaemia has only been reported as a very rare complication of SGLT2i treatment in association with diabetic ketoacidosis (Gajjar & Luthra, 2019).

In contrast, the use of SGLT‐2i in horses is commonly associated with hypertriglyceridaemia (Kellon & Gustafson, 2022; Lindase et al., 2023; Sundra et al., 2022; Sundra, Rossi, et al., 2023). This appears to be mild in most cases, not associated with triglyceride‐induced renal or hepatic dysfunction, and not linked to any treatment‐associated weight loss, but in isolated cases may be severe (Kellon & Gustafson, 2023). Additionally, equine SGLT2i‐associated hypertriglyceridaemia appears to occur predominantly in the short term, is large without apparent short‐term clinical effects and triglyceride concentrations mostly decline with continued therapy (Sundra et al., 2022). The pathophysiology of SGLT2i‐associated hypertriglyceridaemia in the horse has not been elucidated but it may share some similarities with euDKA in people. Further, the differences in the phenomena may relate to the relative importance of hepatic ketogenic pathways in horses compared with people. In people, in response to low plasma glucose, adipose tissues are mobilized and undergo hepatic conversion to ketones such that serum ketone concentrations increase (Long et al., 2021). Whereas in horses, free fatty acids that are mobilized in response to a negative energy balance preferentially undergo hepatic conversion to triglycerides (and lipoproteins) (Naylor et al., 1980; Watson et al., 1991). Equine ketogenic pathways appear relatively unimportant such that ketosis is rare (Rose & Sampson, 1982).

Although equine SGLT2i‐associated hypertriglyceridaemia generally appears to be short‐lived and clinically silent, studies that monitor serum triglyceride concentrations and their impact on organ function over a longer period are lacking. Hypertriglyceridaemia in the horse has previously been associated with both severe (Watson et al., 1992) and minimal/absent clinical effects (Dunkel et al., 2014). The underlying reason for the striking difference in clinical signs associated with hypertriglyceridaemia is not clear. Whilst the plasma lipoprotein profiles in clinically affected hyperlipaemia cases have been well characterized (Watson et al., 1991), further research is required into the plasma lipid profiles of SGLT2i‐associated hypertriglyceridaemia.

Also of note is the fact that there is considerable evidence linking endothelial function and the circulating lipid profile in people (Higashi, 2023) with hypertriglyceridaemia impairing endothelial function (Lewis et al., 1999). Endothelial dysfunction may play a role in the pathogenesis of endocrinopathic laminitis (Gauff et al., 2013; Morgan et al., 2016; Venugopal et al., 2011; Wooldridge et al., 2014) Thus, understanding the effect of SGLT2I and the hypertriglyceridaemia induce on equine endothelial function is vitally important. Additionally, it is important that triglyceride concentrations in horses treated with SGLTI2 are monitored closely and further studies are required to assist in determining the clinical significance of the hypertriglyceridaemia associated with SGLT2i use (Sundra, Lester, et al., 2023).

### 5.2 Other side effects

The other side effects reported to be associated with SGLT2i use in horses are polyuria and polydipsia (PU/PD). Ten out of 51 horses (16%) treated with ertugliflozin exhibited PU/PD (Sundra et al., 2022). Although these horses did not develop signs of dehydration or hypovolaemia, the authors recommended that clinicians should be aware of the potential for volume depletion and ensure free access to ample quantities of water (Sundra et al., 2022). In contrast, PU/PD was not noted in association with velagliflozin therapy (Meier et al., 2019). Whilst SGLT2i have the potential to cause urinary tract infections associated with glucosuria, this has not been reported in horses (Sundra et al., 2022). Additionally, where reported markers of hepatobiliary function remain normal (Meier et al., 2019; Sundra et al., 2022; Sundra, Rossi, et al., 2023).

## 6 TOXICITY OF SGLT2I

To the best of the authors' knowledge there are no pharmacokinetic/pharmacodynamic or toxicity studies in the horse. One study of only two horses (Michanek et al., 2023) used 6‐fold higher doses of canagliflozin (1.8 and 3.6 mg/kg) than have been reported for clinical cases (0.3–0.6 mg/kg) without apparent adverse effects and in a study described above horses received velagliflozin orally at doses 10 times higher (3 mg/kg p.o. sid) than the dose used to prevent laminitis in an experimental trial (0.3 mg/kg p.o. sid) Toxicity studies in laboratory rodents and beagles revealed that very high single (375–1000 mg/kg) and repeated (50–180 mg/kg/day) doses of dapagliflozin were well tolerated (Tirmenstein et al., 2013).

## 7 CONCLUSION

In conclusion, there is a small amount of scientific literature reporting the use of SGLT2i to aid the management of equine ID and consequently reduce the risk of hyperinsulinaemia‐associated laminitis in conjunction with dietary management. However, the doses used are largely extrapolated from human studies with limited consideration of species‐specific variations and to the best of the authors' knowledge no toxicity studies have been performed on the horse. In addition, there is no consideration of the fundamental differences between ID in horses and humans, particularly the fact that most horses with ID remain hyperinsulinaemic but normoglycaemic such that increased urinary loss of glucose may not fully explain the beneficial effects of these drugs. Further study into the potentially deleterious effects of treatment‐associated hypertriglyceridaemia is also required. Finally, the effect of SGLT2i therapy on circulating concentrations of adipokines in horses and consequent laminitis risk has yet to be determined.

## AUTHOR CONTRIBUTIONS

Both authors contributed to the writing of this manuscript.

## CONFLICT OF INTEREST STATEMENT

Nicola Menzies‐Gow has previously served as a consultant for and received payments for educational material from Boehringer Ingelheim. Edward J. Knowles is employed by CVS UK Ltd and provides clinical diagnostic services through Axiom Veterinary Laboratory. He has received payments from Boehringer Ingelheim for educational material.

## ANIMAL AND WELFARE STATEMENT

Not applicable.

## ETHICS STATEMENT

This is not relevant for a review article as no new data was generated.

## Data Availability

Data sharing is not applicable to this article as no new data were created or analysed in this study.
